# Baseline type 2 diabetes had a significant association with elevated high sensitivity cardiac troponin T levels in Chinese community-dwelling population: a 5-year prospective analysis

**DOI:** 10.1186/s12986-017-0229-8

**Published:** 2017-11-25

**Authors:** Shihui Fu, Rongjie Jin, Leiming Luo, Ping Ye

**Affiliations:** 1 Department of Geriatric Cardiology, Chinese People’s Liberation Army General Hospital, Beijing, 100853 China; 2 Department of Cardiology and Hainan Branch, Chinese People’s Liberation Army General Hospital, Beijing, China

**Keywords:** Chinese community-dwelling population, Fasting blood glucose, High sensitivity cardiac troponin T, Postprandial blood glucose, Type 2 diabetes

## Abstract

**Background:**

The present analysis was designed to investigate the association of type 2 diabetes (T2D) with high sensitivity cardiac troponin T (hs-cTnT) during the 5 years of follow-up, and explore which one of fasting blood glucose (FBG) and postprandial blood glucose (PBG) is a determinant of this association in Chinese community-dwelling population.

**Methods:**

This prospective community-based analysis was conducted based on 730 participants without coronary artery disease and hs-cTnT values ≥14 pg/mL receiving two measurements of hs-cTnT levels at baseline and follow-up of 5 years.

**Results:**

Prevalence of T2D was 16.2% (118 participants). Median hs-cTnT levels were 4 (3-7) pg/mL and 6 (5-9) pg/mL at baseline and follow-up, respectively. The variation in hs-cTnT levels had a median of 2 (0-4) pg/ml (*p* < 0.001 for variation), and incidence of hs-cTnT levels ≥14 pg/ml was 7.1% (52 participants) at follow-up. T2D had a significant association with elevated hs-cTnT levels in multivariate Logistic regression models (*p* < 0.05). Elevated levels of PBG (*p* < 0.05) rather than FBG (*p* > 0.05) determined the significant association with elevated hs-cTnT levels in multivariate linear regression models.

**Conclusions:**

This community-based analysis observed that there was a significant increment of hs-cTnT levels, and baseline T2D had a significant association with elevated hs-cTnT levels during the 5 years of follow-up. Moreover, the present analysis demonstrated that PBG rather than FBG played a crucial role in this association in Chinese community-dwelling population without CAD.

**Electronic supplementary material:**

The online version of this article (10.1186/s12986-017-0229-8) contains supplementary material, which is available to authorized users.

## Background

Coronary artery disease (CAD) is a significant cause of death in community-dwelling population, and cardiac troponin T is a well-established biomarker providing the diagnostic and prognostic information in patients with CAD. CAD is very common in patients with type 2 diabetes (T2D), and cardiac troponin T is a big signal but not specific for CAD in patients with T2D. T2D has a significant association with cardiac troponin T measured with the conventional assay in patients with CAD [1]. However, the conventional assay is of limited use in patients without CAD, because they generally do not have detectable levels of cardiac troponin T using the conventional assay [2]. Recently, novel high sensitivity assay for cardiac troponin T with nearly ten times the detection limit of conventional assay has become available to further improve the diagnostic and prognostic performance of cardiac troponin T measured with the conventional assay [3–5]. Limited studies have probed the variation in high sensitivity cardiac troponin T (hs-cTnT) levels as well as the association of T2D with elevated hs-cTnT levels in community-dwelling population inclusive of patients with CAD. Previous cross-sectional and short-term studies have demonstrated the relationships of hs-cTnT with cardiac structure and cardiovascular mortality, but been unable to identify the slight variation in hs-cTnT levels and analyze the association of T2D with elevated hs-cTnT levels [6, 7]. Long-term follow-up is needed to clarify whether T2D had a significant association with elevated hs-cTnT levels. Moreover, large-scale data from community-dwelling population rather than clinical patients are scarce, and most of published studies in community-dwelling population have included few Chinese [6, 7]. Small-scale studies on clinical patients restrict the interpretation of relationship between T2D and hs-cTnT in community-dwelling population, and results from western studies can not be directly taken to suggest the association T2D with elevated hs-cTnT levels in China [1, 2]. In this prospective analysis of Chinese community-dwelling population without CAD, the follow-up of 5 years allowed us to have two measurements of hs-cTnT levels, and finally obtain the variation in hs-cTnT levels. Additionally, the present analysis attempted to investigate the association of baseline T2D with elevated hs-cTnT levels during the 5 years of follow-up, and explore which one of fasting blood glucose (FBG) and postprandial blood glucose (PBG) is a determinant of this association in Chinese community-dwelling population.

## Methods

### Study population

This prospective analysis was conducted based on 1680 community-dwelling residents with the age of 18 years or more voluntarily participating in a large medical check-up program at the health service centers in Beijing, China, from May 2007 to July 2009. In the first stage of sampling, three districts (Fengtai, Shijingshan and Daxing) were selected from 18 districts in Beijing. In the second stage of sampling, four communities were selected from these districts. In the third stage of sampling, participants were selected from these communities. The follow-up visit was conducted from February 2013 to September 2013. There were 181 participants lost, and follow-up of 5 years was completed for 1499 participants. Exclusion criteria were CAD [189 participants, diagnosed by chief physicians with history, symptoms of typical angina, cardiac markers and tests, such as electrocardiogram (resting/exercise), echocardiogram, radionuclide imaging, computed tomography and coronary arteriography, according to American College of Cardiology/American Heart Association/European Society of Cardiology guidelines], hs-cTnT levels ≥14 pg/mL (137 participants), missing values for variables (430 participants) and death (52 participants) [8–11]. Since the 99th percentile of hs-cTnT levels in normal population is 14 pg/mL, the cut-point 14 pg/mL is recommended as diagnostic criteria of elevated hs-cTnT levels by standard specification of manufacturers. Remaining 730 participants had two measurements of hs-cTnT levels at baseline and follow-up. Baseline characteristics of participants were comparable before and after exclusion (Additional file 1: Table S1).

### Physical examination

Physical examination was conducted by trained physicians. Weight was measured with a digital scale, and height was measured with a wall-mounted measuring tape, with light clothes and no shoes. Body mass index (BMI) was calculated as weight (kg) divided by squared height (m^2^). Two measurements of blood pressure were taken with a standard mercury sphygmomanometer (Yuwell Medical Equipment & Supply Co., Ltd., Jiangsu, China) from the right arm of participants in the seated position, and systolic and diastolic blood pressures (SBP and DBP) were recorded as an average level of two measurements at intervals of at least 1 min.

### Laboratory test

Participants were fasting at least 12 h at the time of blood sample collection, and test was conducted by qualified technicians unaware of patients’ characteristics in our central laboratory. Samples were stored at −80 °C and quantified using an Elecsys Troponin T high sensitive assay (Roche Products Ltd., Basel, Switzerland) on a Modular Analytics E170 autoanalyzer (Roche Products Ltd., Basel, Switzerland) by an electrochemiluminescence immunoassay method. Based on the standard specification of manufacturers, the lower limit of the high-sensitivity assay is 3 pg/mL, whereas that of the conventional assay is 100 pg/mL (colloidal gold labeled method) or 10 pg/mL (chemiluminescence method). Concentrations of FBG, triglyceride (TG), high-density lipoprotein cholesterol (HDL-c) and low-density lipoprotein cholesterol (LDL-c) and uric acid were determined using the Roche enzymatic assay (Roche Products Ltd., Basel, Switzerland) on a Roche autoanalyzer (Roche Products Ltd., Basel, Switzerland). For participants receiving the collection of fasting blood sample, standard oral glucose tolerance test was conducted subsequently at timed intervals of 2 h after drinking 75 g glucose load.

### Diagnostic criteria

Diagnostic criteria of hypertension were an average SBP ≥ 140 mmHg, an average DBP ≥ 90 mmHg, and/or an use of anti-hypertensive medications. Diagnostic criteria of T2D were FBG ≥ 7.0 mmol/L, PBG ≥ 11.1 mmol/L and/or use of insulin or oral hypoglycemic medications. High TG was diagnosed as TG ≥ 2.26 mmol/L (200 mg/dl), low HDL-c as HDL-c < 1.04 mmol/L (40 mg/dL), and high LDL-c as LDL-c ≥ 4.14 mmol/L (160 mg/dL), and/or use of hypolipidemic medications. Hyperuricaemia was diagnosed as the levels of uric acid more than 360 μmol/L in women and more than 420 μmol/L in men [12].

### Statistical analysis

Continuous variables with normal distribution were reported as mean with standard deviation, and continuous variables with skewed distribution were reported as median with interquartile range. Categorical variables were reported as number with proportion. Comparison between groups was conducted by Student’s t-test (continouous variables with normal distribution), Mann–Whitney U test (continouous variables with skewed distribution) and Chi-square test (categorical variables). Bivariate correlation was derived from Pearson and Spearman correlations. Wilcoxon signed-rank test was applied to compare the hs-cTnT levels at baseline and follow-up. Logistic regression analysis was applied in two models. In the first model to include age and gender as adjustment variables, and in the second model the presence of age, gender, obesity, hypertension, high TG, low HDL-c, high LDL-c and hyperuricemia. The variation in hs-cTnT levels was transformed to meet the multinormality assumption, and then linear regression analysis was applied in two models. In the first model to include age and gender as adjustment variables, and in the second model the presence of age, gender, body mass index, SBP, DBP, TG, HDL-c, LDL-c and uric acid. Two sided *p* < 0.05 was considered to indicate the statistical significance. Statistical analysis was conducted with Statistical Package for the Social Science (SPSS) software (version 17.0; SPSS Inc., Chicago, IL, USA).

## Results

For all participants, median age was 59 (range: 25-89) years, and proportion of males was 42.3% (309 participants). Prevalence of T2D was 16.2% (118 participants). Median hs-cTnT levels were 4 (3-7) pg/mL and 6 (5-9) pg/mL at baseline and follow-up, respectively (*p* < 0.001 for variation). Incidence of hs-cTnT levels ≥14 pg/ml was 7.1% (52 participants) at follow-up. Not only age, SBP and PBG levels were significantly higher, and HDL-c levels were significantly lower, but also proportions of age ≥ 60, males, low HDL-c and T2D were significantly higher in participants with hs-cTnT levels ≥14 pg/ml than those with hs-cTnT <14 pg/ml at follow-up (*p* < 0.05 for all; Table 1). The variation in hs-cTnT levels with a median of 2 (0-4) pg/ml was related to not only levels of age, SBP, TG, uric acid and PBG, but also proportions of age ≥ 60, males, hypertension, hyperuricemia and T2D (p < 0.05 for all; Table 1).

**Table 1:** Characteristics of participants divided by hs-cTnT levels <14 or ≥14 pg/ml at follow-up

Characteristics	Follow-up hs-cTnT < 14 pg/ml (*n* = 678)	Follow-up hs-cTnT ≥ 14 pg/ml (*n* = 52)	*P* value^*^	*P* value^†^
At baseline				
Age (year)	58(50-67)	72(64-75)	<0.001	<0.001
Age ≥ 60 (%)	317(46.8)	45(86.5)	<0.001	<0.001
Males (%)	274(40.4)	35(67.3)	<0.001	0.009
BMI (kg/m^2^)	25.27(23.19-27.59)	25.37(23.39-27.77)	0.703	0.074
Obesity (%)	145(21.4)	11(21.2)	0.969	0.295
SBP (mmHg)	126(114-140)	131(121-144)	0.034	<0.001
DBP (mmHg)	77(70-83)	77(70-86)	0.923	0.323
Hypertension (%)	302(44.5)	29(55.8)	0.117	<0.001
TG (mmol/L)	1.47(1.05-2.15)	1.49(1.18-2.11)	0.788	0.020
High TG (%)	153(22.6)	8(15.4)	0.229	0.587
HDL-c (mmol/L)	1.36(1.15-1.59)	1.27(1.00-1.47)	0.005	0.131
Low HDL-c (%)	88(13.0)	14(26.9)	0.005	0.309
LDL-c (mmol/L)	2.92(2.47-3.30)	3.02(2.47-3.37)	0.637	0.690
High LDL-c (%)	42(6.2)	1(1.9)	0.207	0.430
Uric acid (μmol/L)	276(232-332)	305(248-338)	0.063	<0.001
Hyperuricemia (%)	46(6.8)	4(7.7)	0.803	0.010
FBG (mmol/L)	4.95(4.51-5.45)	4.94(4.38-5.77)	0.897	0.835
PBG (mmol/L)	6.34(5.23-8.30)	7.57(5.72-10.95)	0.006	<0.001
T2D (%)	99(14.6)	19(36.5)	<0.001	0.002
hs-cTnT (pg/ml)	4(3-6)	9(6-12)	<0.001	<0.001
At follow-up				
hs-cTnT (pg/ml)	6(5-8)	16(14-20)	<0.001	<0.001

^*^Comparison between groups defined by hs-cTnT levels <14 pg/mL or ≥14 pg/mL at follow-up; ^†^Bivariate correlation of all variables with the variation in hs-cTnT levels

*Abbreviations*: *hs-cTnT* high sensitivity cardiac troponin T, *BMI* body mass index, *SBP* systolic blood pressure, *DBP* diastolic blood pressure, *TG* triglyceride, *HDL-c* high density lipoprotein cholesterol, *LDL-c* low density lipoprotein cholesterol, *FBG* fasting blood glucose, *PBG* postprandial blood glucose, *T2D* type 2 diabetes

Compared with those without T2D, participants with T2D had not only significantly higher percentages of age ≥ 60, obesity, high TG and low HDL-c, but also higher age, BMI, SBP, TG, FBG and PBG levels, and lower HDL-c levels, at baseline, as well as higher hs-cTnT levels at baseline and follow-up (Table 2). T2D had a significant association with elevated hs-cTnT levels in the first and second models (*p* < 0.05; Table 3). Elevated levels of PBG (p < 0.05) rather than FBG (*p* > 0.05) determined the significant association with elevated hs-cTnT levels in the first and second models (Table 4).

**Table 2:** Characteristics of participants with and without T2D at baseline

Characteristics	With T2D (*n* = 118)	Without T2D (*n* = 612)	*P* value
At baseline			
Age (year)	64(56-72)	58(50-67)	<0.001
Age ≥ 60 (%)	78(66.1)	284(46.4)	<0.001
Males (%)	56(47.5)	253(41.3)	0.218
BMI (kg/m^2^)	26.16(23.87-28.72)	25.15(23.16-27.34)	0.008
Obesity (%)	39(33.1)	117(19.1)	0.001
SBP (mmHg)	131(118-143)	126(113-140)	0.010
DBP (mmHg)	79(70-82)	77(70-83)	0.566
Hypertension (%)	63(53.4)	268(43.8)	0.055
TG (mmol/L)	1.85(1.25-2.55)	1.44(1.02-2.07)	<0.001
High TG (%)	40(33.9)	121(19.8)	0.001
HDL-c (mmol/L)	1.19(1.05-1.39)	1.37(1.18-1.62)	<0.001
Low HDL-c (%)	24(20.3)	78(12.7)	0.029
LDL-c (mmol/L)	2.98(2.38-3.35)	2.92(2.47-3.30)	0.611
High LDL-c (%)	9(7.6)	34(5.6)	0.382
Uric acid (μmol/L)	282(232-349)	278(234-331)	0.625
Hyperuricemia (%)	11(9.3)	39(6.4)	0.245
FBG (mmol/L)	6.69(5.47-8.10)	4.87(4.44-5.24)	<0.001
PBG (mmol/L)	12.9(9.70-16.46)	5.96(5.06-7.30)	<0.001
hs-cTnT (pg/ml)	5(3-9)	4(3-6)	0.001
At follow-up			
hs-cTnT (pg/ml)	8(6-11)	6(5-8)	<0.001

*Abbreviations*: *T2D* type 2 diabetes, *BMI* body mass index, *SBP* systolic blood pressure, *DBP* diastolic blood pressure, *TG* triglyceride, *HDL-c* high density lipoprotein cholesterol, *LDL-c* low density lipoprotein cholesterol, *FBG* fasting blood glucose, *PBG* postprandial blood glucose, *hs-cTnT* high sensitivity cardiac troponin T

**Table 3:** Logistic regression model to analyze the association of T2D with hs-cTnT

Characteristics	HR	95%CI	*P* value
First model^a^	2.473	1.292-4.732	0.006
Second model^b^	2.492	1.270-4.892	0.008

^a^First model: adjusted for age and gender; ^b^Second model: adjusted for age, gender, obesity, hypertension, high triglyceride, low high density lipoprotein cholesterol, high low density lipoprotein cholesterol and hyperuricemia

*Abbreviations*: *T2D* type 2 diabetes, *hs-cTnT* high sensitivity cardiac troponin T, *HR* hazard ratio, *CI* confidence interval

**Table 4:** Linear regression model to analyze the association of FBG and PBG with hs-cTnT

Characteristics	FBG			PBG		
	Effect	SE	*P* value	Effect	SE	*P* value
First model^a^	0.037	0.022	0.262	0.075	0.009	0.027
Second model^b^	0.034	0.022	0.329	0.072	0.009	0.038

^a^First model: adjusted for age and gender; ^b^Second model: adjusted for age, gender, body mass index, systolic blood pressure, diastolic blood pressure, triglyceride, high density lipoprotein cholesterol, low density lipoprotein cholesterol and uric acid

*Abbreviations*: *FBG* fasting blood glucose, *PBG* postprandial blood glucose, *hs-cTnT* high sensitivity cardiac troponin T, *SE* standard error

## Discussion

In Chinese community-dwelling population, the present analysis observed that there was a significant increment of hs-cTnT levels, and baseline T2D had a significant association with elevated hs-cTnT levels during the 5 years of follow-up. Moreover, the present analysis demonstrated that PBG but not FBG played a crucial role in this association in Chinese community-dwelling population without CAD.

As is well known, cardiac troponin T has clearly diagnostic and prognostic value in patients with CAD [13, 14]. Mechanisms of cardiac troponin T elevation include the mismatch between oxygen supply and demand, inflammation, strain and apoptosis [15, 16]. Elevated levels of cardiac troponin T reflect the pathophysiological process of cardiovascular damage, and represent the rising morbidity and mortality of CAD. Compared with the conventional assay, novel high-sensitivity assay of cardiac troponin T had superior ability to improve the diagnosis of CAD and predict the outcome of CAD [17, 18]. Thus, hs-cTnT is a significant biomarker to facilitate the early identification of CAD and define the risk of CAD in community-dwelling population.

CAD is very common in patients with T2D, and cardiac troponin T is a big signal but not specific for CAD in patients with T2D. Although T2D is a main factor affecting the cardiac troponin T measured with the conventional assay in clinical patients, the association of T2D with cardiac troponin T elevation measured with recently emergent high-sensitivity assay has not been explicit in community-dwelling population [1, 2]. Large-scale data from community-dwelling population rather than clinical patients are scarce, and most of published studies in community-dwelling population have included few Chinese [6, 7]. Small-scale studies on clinical patients restrict the interpretation of relationship between T2D and hs-cTnT in community-dwelling population, and results from western studies can not be directly taken to suggest the association of T2D with elevated hs-cTnT levels in China [1, 2]. Meanwhile, previous cross-sectional and short-term studies have demonstrated the relationships of hs-cTnT with cardiac structure and cardiovascular mortality, but been unable to identify the slight variation in hs-cTnT levels and analyze the association of T2D with elevated hs-cTnT levels [6, 7]. Long-term follow-up is needed to clarify whether T2D had a significant association with elevated hs-cTnT levels. Two measurements of hs-cTnT levels at baseline and follow-up of 5 years offered the opportunity for detecting the slight variation in hs-cTnT levels and evaluating the association of T2D with elevated hs-cTnT levels. The present analysis manifested a significant increment in hs-cTnT levels, and extended a significant association of baseline T2D with elevated hs-cTnT levels to Chinese community-dwelling population, reminding us that Chinese community-dwelling population without CAD have chronically elevated cardiac troponin T levels with the high sensitivity assay. Even slight alteration in cardiac troponin T levels is indicative of cardiovascular damage [19]. Moreover, results from the present analysis illustrated that cardiac troponin T was chronically elevated in response to T2D, and T2D accumulated the risk of cardiovascular damage in Chinese community-dwelling population without CAD.

Previous studies have failed to decide which one of FBG and PBG is a determinant of the association of T2D with elevated hs-cTnT levels in Chinese community-dwelling population without CAD. The present analysis underlined that the association of T2D with elevated hs-cTnT levels was attributed to PBG rather than FBG during the 5 years of follow-up. In other words, there was a significant role of elevated PBG levels in cardiovascular damage, and it should give priority to glycemic control after meals in Chinese community-dwelling population. However, the present paper have several limitations including a lack of full adjustment and residual confounding. Although the present analysis was adjusted for age, gender, body mass index, SBP, DBP, TG, HDL-c, LDL-c and uric acid, one possibility can not be ruled out that there are additional factors influencing hs-cTnT levels and these.potential confounders were not adjusted in the present analysis.

## Conclusions

This community-based analysis observed that there was a significant increment of hs-cTnT levels, and baseline T2D had a significant association with elevated hs-cTnT levels during the 5 years of follow-up. Moreover, the present analysis demonstrated that PBG rather than FBG played a crucial role in this association in Chinese community-dwelling population without CAD.

## Additional file

Additional file 1: Table S1. Characteristics of participants before and after exclusion at baseline. (DOC 25 kb)

## Abbreviations

CAD: Coronary artery disease
DBP: Diastolic blood pressure
FBG: Fasting blood glucose
HDL-c: Highdensity lipoprotein cholesterol
hs-cTnT: High sensitivity cardiac troponin T
LDL-c: Low-density lipoprotein cholesterol
PBG: Ostprandial blood glucose
SBP: Systolic blood pressure
T2D: Type 2 diabetes
TG: Triglycerides
