# Supplementary material for: Baseline type 2 diabetes had a significant association with elevated high sensitivity cardiac troponin T levels in Chinese community-dwelling population: a 5-year prospective analysis
Source: Nutr Metab (Lond). 2017 Nov 25;14:73. doi: 10.1186/s12986-017-0229-8 (PMC5702227; doi:10.1186/s12986-017-0229-8)
Supplement: Additional file 1: Table S1. — Characteristics of participants before and after exclusion at baseline. (DOC 25 kb) [file 12986_2017_229_MOESM1_ESM.doc]

**Additional file 1: Table S1. Characteristics of participants before and after exclusion at baseline**

| **Characteristics** | **Before exclusion**  **(n=1499)** | **After exclusion**  **(n=730)** | **P value**^＊^ |
| --- | --- | --- | --- |
| Age (year) | 58(50-68) | 59(51-68) | 0.568 |
| Males (%) | 309(42.3) | 309(42.3) | 1.000 |
| BMI (kg/m^2^) | 25.24(23.31-27.55) | 25.28(23.22-27.62) | 0.544 |
| SBP (mmHg) | 127(116-140) | 127(115-140) | 0.606 |
| DBP (mmHg) | 77(70-83) | 77(70-83) | 0.747 |
| TG (mmol/L) | 1.46(1.05-2.13) | 1.47(1.06-2.13) | 0.544 |
| FBG (mmol/L) | 4.99(4.56-5.55) | 4.95(4.51-5.46) | 0.168 |

**Abbreviations:** BMI: body mass index; SBP: systolic blood pressure; DBP: diastolic blood pressure; TG: triglyceride; FBG: fasting blood glucose.
